# Preparation of TiO_2_ Anatase Nanocrystals by TiCl_4_ Hydrolysis with Additive H_2_SO_4_

**DOI:** 10.1371/journal.pone.0021082

**Published:** 2011-06-15

**Authors:** Wenbing Li, Tingying Zeng

**Affiliations:** 1 Department of Chemistry and Biology, Jackson State University, Jackson, Mississippi, United States of America; 2 The Center for Excitonics, Massachusetts Institute of Technology, Cambridge, Massachusetts, United States of America; Massey University, New Zealand

## Abstract

A new methodology was developed to synthesize uniform titania anatase nanocrystals by the hydrolysis of titanium chloride in sulfuric acid aqueous solutions at 0–90°C. The samples were characterized by Raman spectroscopy, UV-visible spectroscopy, transmission electron microscopy (TEM), electron diffraction (ED), and an Energy dispersive X-ray spectroscopy (EDS). The effects of the reaction temperature, mole ratio of SO_4_^2−^ to Ti^4+^, and the calcinations temperature on the particle size and crystal phase were investigated. Depending on the acidity, the hydrolysis temperature, and the calcination temperature, rhombic anatase nanocrystals sizes in the range of 10 nm to 50 nm were obtained. In the additive of sulfuric acid, Raman spectra and electron diffraction confirmed that the nanoparticles are composed of anatase TiO_2_. No other titania phases, such as rutile or brookite, were detected.

## Introduction

It is well known that TiO_2_ occurs in nature in three distinct crystallographic phases: anatase, rutile, and brookite. While anatase TiO_2_ are the most widely used photocatalysts for oxidative decomposition of organic compounds, and an excellent photocatalyst for photodecomposition and solar-energy conversion due to its high photoactivity.[1] It has the advantages of both cheapness and nontoxicity, in addition to its excellent functionality and long-term stability. The configurations of titanium oxide researched and reported have mainly been powders or films based on materials.[2]–[4] A notable problem connected with these traditional preparations is that the growth of TiO_2_ nanocrystallites take a long time. Therefore it is highly desirable to find some new ways that are capable of overcoming the above problems to prepare crystal structure TiO_2_. Many attempts have been made in this field over the past few years.[5]–[16] At the same time, recent photocatalytic studies have demonstrated that the photoactivity of anatase nanoparticles is strongly particle size dependent.[17], [18] The applications for TiO_2_ are also strongly dependent on the crystalline structure and morphology.[10], [16]–[18] Thus, it is very important to develop synthetic methods in which the crystalline form. It is also important that the TiO_2_ sizes and shapes be controlled.[19] Anatase nanoparticles have been synthesized primarily by solution chemistries involving titanium sulfates and organic titanium.[20]–[27] These methods have shortcomings since chemical impurities or minor accessory phases are always present in the final products. In the case of organic hydrolytic reactions, TiO_2_ nanoparticles obtained are crystallized primarily in the anatase phase but a minor phase of brookite couldn't be eliminated by tuning the reaction conditions.[28]–[42] The presence of trace amounts of brookite might have side effects on the application of anatase nanoparticles in photocatalytic reactions and many other chemical processes. Hydrolysis of TiCl_4_ has extensively been reported for the synthesis of anatase nanocrystals, however, the preparation of titania anatase nanocrystals directly in sulfuric acid solution is seldom reported.

In this study, the preparation of titania anatase nanocrystals by hydrolysis of TiCl_4_ with diluted sulfuric acid solution was studied. With the precisely control of reaction parameters, we could get titania anatase nanocrystals and achieve high phase purity anatase. To the best of our knowledge, such works have not yet been reported. The effects of the reaction temperature, sulfuric acid concentration, and the calcination temperature on the particle size and crystal phase were investigated.

## Materials and Methods

### 1). Materials

All chemicals were obtained commercially and used without further purification. Titanium chloride (TiCl_4_, 99.90%) and Concentrated Sulfuric acid (98%) were obtained from Fisher. Concentrated NH_3_.H_2_O was purchased from Sigma-Aldrich. All chemicals, unless specified, were of reagent grade. Deionized (DI) water, with a resistivity greater than 18.0 MΩ·cm (Millipore Milli-Q system), was used in preparing the aqueous solutions. All glassware used in the experiments was washed with freshly prepared aqua regia and rinsed thoroughly in tap water first and then DI water before using.

### 2). Preparation of TiO2 anatase nanocrystals

**Figure 1:**
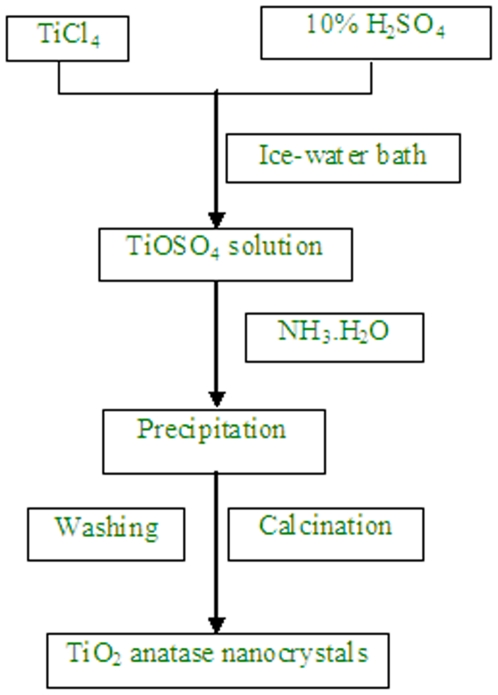
Process of TiO_2_ anatase nanocrystals preparation.

In the procedure, TiCl_4_ was used as a main starting material. The detail process is shown in Figure 1. In brief, 1ml TiCl_4_ was slowly added to different amounts of diluted sulfuric acid (10%) solution at 0°C in an ice-water bath with vigorously stirring. During the mixing process, white fume, presumably HCl, was released as a consequence of the hydrolysis of TiCl_4_ with water. After about half an hour, a grey solution was formed with continuous stirring. Then when the solution was heated to above 60°C, it became clear solution. And then the solution was kept at room temperature or heated at different temperatures for one hour. Later, concentrated NH_3_.H_2_O was added drop by drop to the solution until the pH value reached about 7. During the process of adding concentrated NH_3_.H_2_O, the color of solution changed to white. At last, the white solution was cooled to room temperature and gelled 12 hours. The hydrous TiO_2_ powders were filtered out and washed with DI water until there was no white sediment with 0.1 M AgNO_3_ solution, and then dried at room temperature in a vacuum oven. In some cases, the dried TiO_2_ powders were calcinated at 400°C and 600°C for two hours separately, both producing an off-white powders.

### 3). Raman spectroscopy

Raman spectrometer was used for crystal phase identification. Raman spectroscopy is a form of vibrational spectroscopy, much like infrared (IR) spectroscopy. It exhibits high specificity and is compatible with aqueous and solid systems. No special preparation of the sample is needed, and the timescale of the experiment is short. Raman spectrum analysis was conducted using a Raman System's R-3000 spectrometer with a solid-state diode laser operating at 532 nm. The Raman system's incident power is 25 mW, which has a wavelength range of ∼200–4000 cm^−1^.

### 4). Extinction spectra

The extinction spectra were recorded on a Shimadzu UV-2101 Spectrophotometer (Shimadzu Corporation Japan) using a 1-cm path length quartz cuvette at room temperature and the spectra were recorded in the range 200–800 nm.

### 5). TEM and ED measurements

A drop of well-sonicated solution containing the nanoparticles was deposited onto a 400 mesh Cu grids with supporting carbon film. (Electron Microscopy Sciences, PA). The samples were allowed to dry at room temperature overnight. A JEOL 100CX electron microscope operated at 100 KV was used to obtain the TEM images and ED spectra.

### 6). Energy dispersive X-ray spectroscopy (EDS)

EDS is an analytical technique used for the elemental analysis or chemical characterization of a sample. The chemical compositions of the ultrafine nanoparticles were determined by using a JEOL 5400LV equipped with Sigma Microanalyzer Level LPX1 Energy Dispersive X-ray Spectrometer (EDS).

## Results and Discussion

When TiCl_4_ hydrolyses in diluted sulfuric acid solution, because of the presence of a certain amount of hydrogen ions, the reaction rate may be slower than in pure water. The reaction process can be described as equation 1: 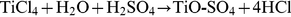
(1)


Then the adding of concentrated NH_3_.H_2_O made the solution becoming white precipitate, and it could lead to the reaction (2):
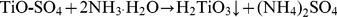
(2)


After the filtering, washing and heating, here is the decomposition of H_2_TiO_3_.
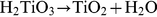
(3)


Table 1 shows a summary of the sample's preparation conditions. The anatase and rutile phases of the prepared samples can be sensitively identified by Raman spectroscopy based on their Raman spectra. All Raman spectra were recorded at room temperature. Consequently, the presence of adsorbed water hindered the study of the hydroxyl groups from these analyses.

**Table 1:** The Comparison of Samples Prepared under Various Hydrolysis Conditions.

Sample No.	H_2_O/TiCl_4_ volume ratio	H_2_SO_4_/TiCl_4_ mole ratio	Hydrolysis T/°C
1	15∶1	4∶1	85
2	10∶1	4∶1	85
3	10∶1	2∶1	85
4	10∶1	2∶1	75
5	10∶1	1∶1	65
6	10∶1	1∶1	25

**Figure 2:**
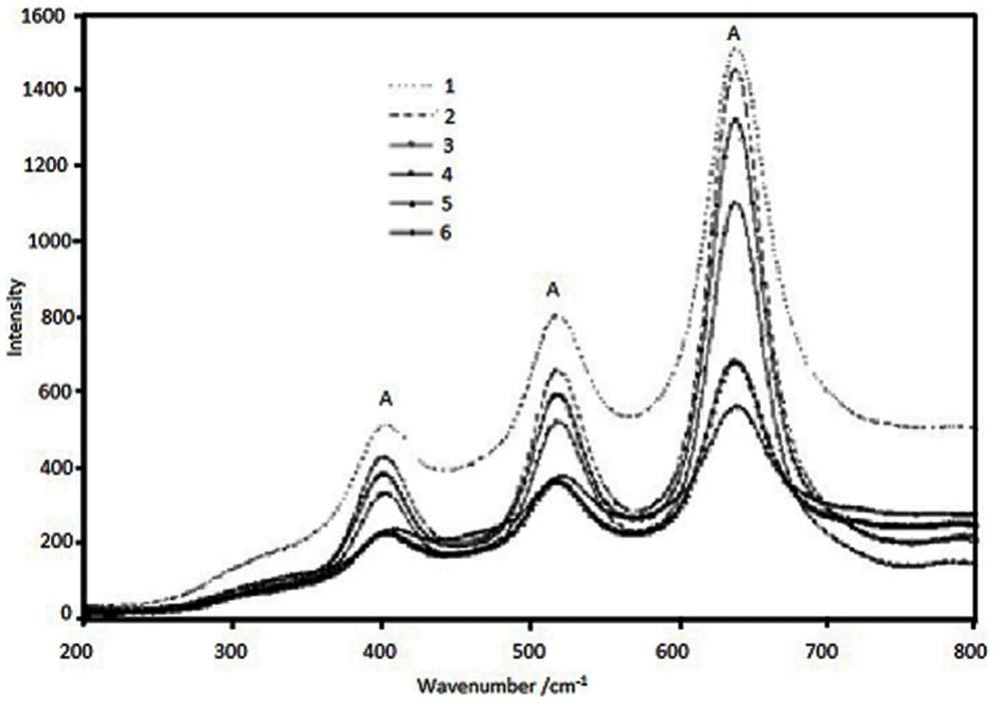
Comparison of the Raman spectra of the samples after calcinations at 400°C: sample 1, sample 2, sample 3, sample 4, sample 5, sample 6.

**Figure 3:**
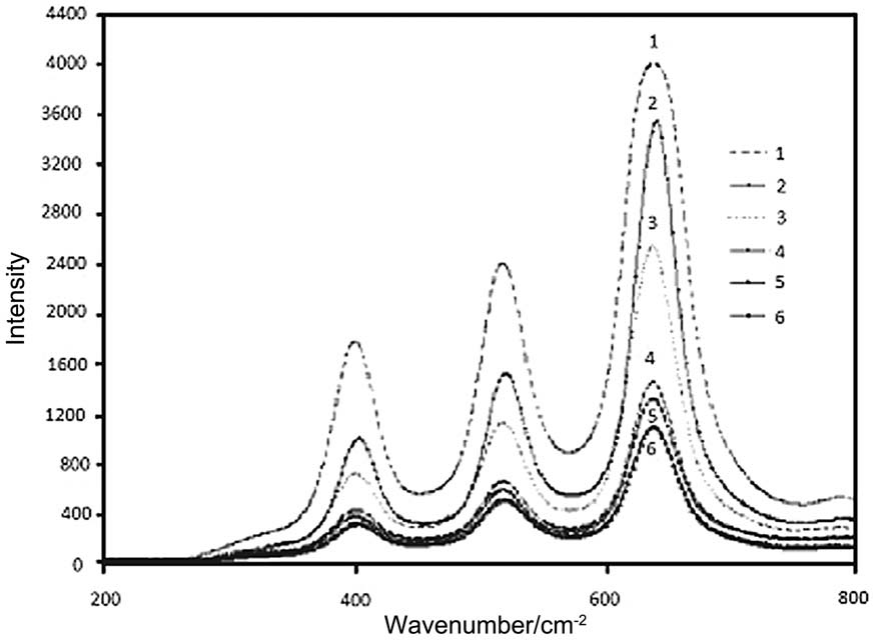
Comparison of the Raman spectrum of the samples after calcinations at 600°C: sample 1, sample 2, sample 3, sample 4, sample 5, sample 6.

Figure 2 and 3 showed the comparison of the Raman spectra of the samples at different reaction conditions. Because of the wavelength range limitation of Raman system R-3000, there is no Raman bands at 144 and 197 cm^−1^. But the anatase phase shows major Raman bands at 399, 515, 519 (superimposed with the 515 cm^−1^ band), and 639 cm^−1^. [37], [38] So samples are in the anatase phase because only characteristic bands (399, 515, 519, and 638 cm^−1^) due to anatase phase are observed. These bands can be attributed to the four Raman-active modes of anatase phase with the symmetries of *B*_1g_, *E*g, *E*g, and *E*g respectively. Obviously, Raman spectra show that the titania sample is in the anatase phase without rutile or brookite. The presence of sulfuric acid is quite effective in promoting the formation of the anatase phase. It can be seen that with the higher sulfuric acid concentration in the system, the higher crystallinity it is. At the same time, with the increase of calcinations temperature, the crystallinity increased. The higher water ratio also increased the crystallinity.

**Figure 4:**
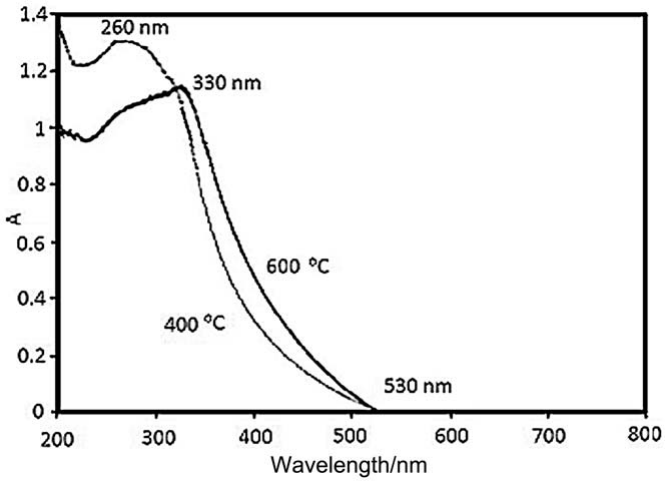
Extinction spectra of sample 1 calcinated at 400 and 600°C.

Figure 4 showed the UV-vis diffuse reflectance spectra of the titania samples calcinated at 400 and 600°C. This absorbance increase shifts to slightly higher wavelengths as the temperature of the powder thermal treatment increases. This phenomenon is more clearly evident by the evolution of the position of the maximum in the derivative of the UV spectra, which corresponds to the inflection point on the spectrum. For the anatase phase, the absorption band edge can be estimated to be around 530 nm, respectively. It clearly shows that titania has strong electronic absorption in the UV region.

**Figure 5:**
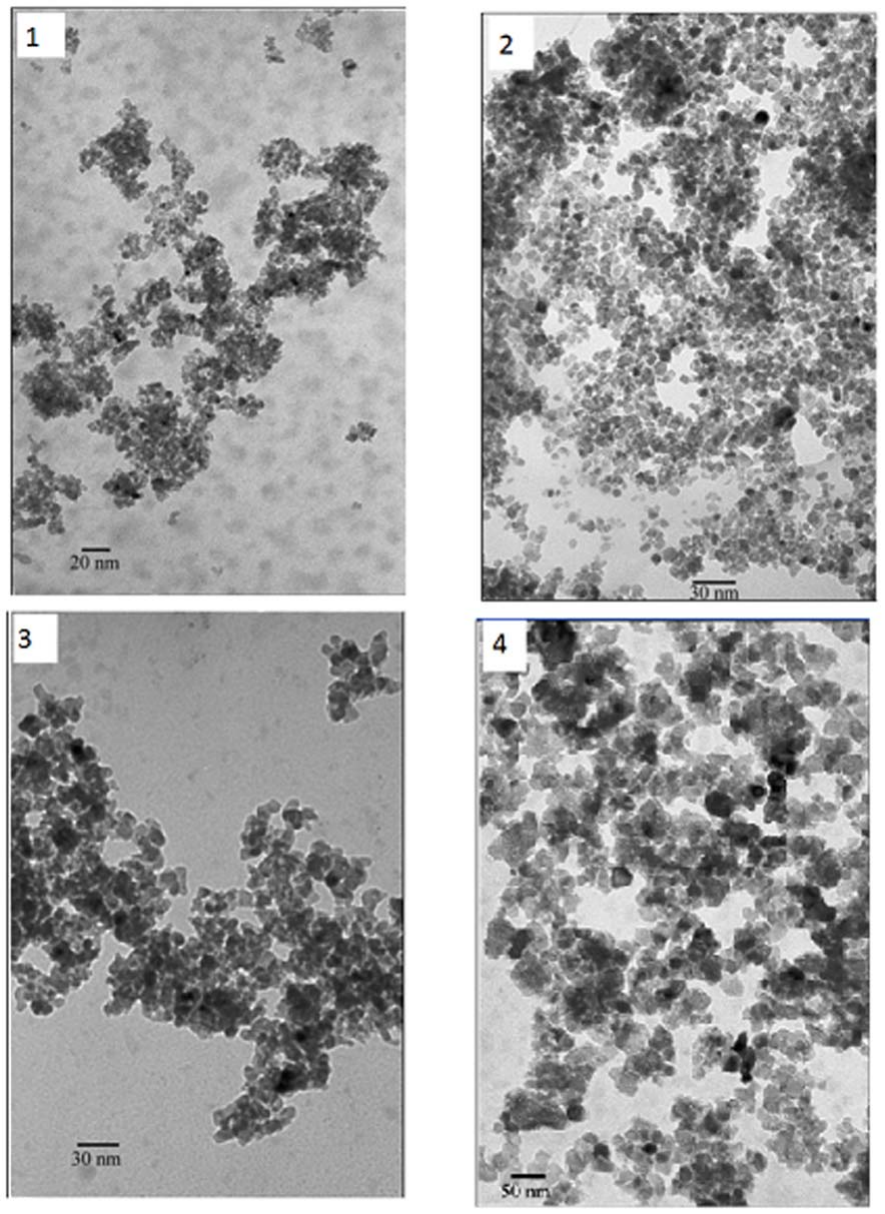
TEM image of the samples calcinated at 400°C for 2 hours: sample 1, sample 2, sample 3, sample 4.

When the samples prepared with sulfuric acid solution were calcinated at 400°C for 2 hours, with the increased sulfuric acid content, the intensity of Raman patterns as well as ED patterns increased. In Figure 5, TEM image of sample 1, sample 2, sample 3 and sample 4 calcinated at 400°C for 2 hours are reported. It showed that the morphology of the samples is close to sphere. The primary particle size of sample 1 is finer than the rest. It can be noted that the primary particle's size in sample 1 is between 6 and 9 nm and the secondary ones are less than 5 nm. After calcinated at 400 and 600°C, most of the particles in the samples exhibit diameters in the range of 10 to 50 nm. With the increasing of hydrolysis temperature, the average size of the nanocrystals decreased. With an increase in water content, the average size of the nanocrystals decreased. The higher sulfuric acid content also decreased the particle size.

**Figure 6:**
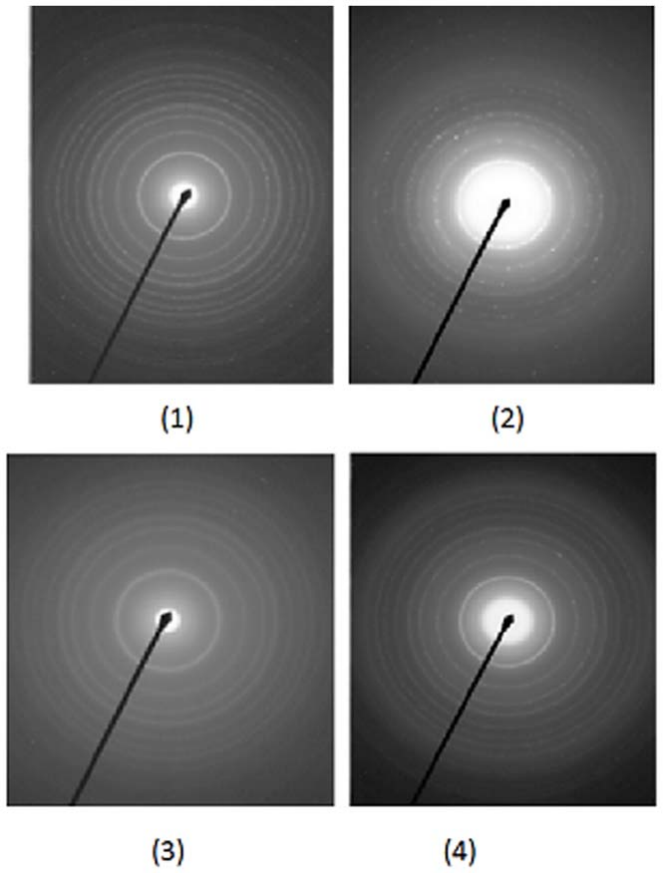
ED images of the samples, (1) sample 1 calcinated at 400°C, (2) sample 2 calcinated at 400°C, (3) sample 1 calcinated at 600°C, (4) sample 2 calcinated at 600°C.

**Figure 7:**
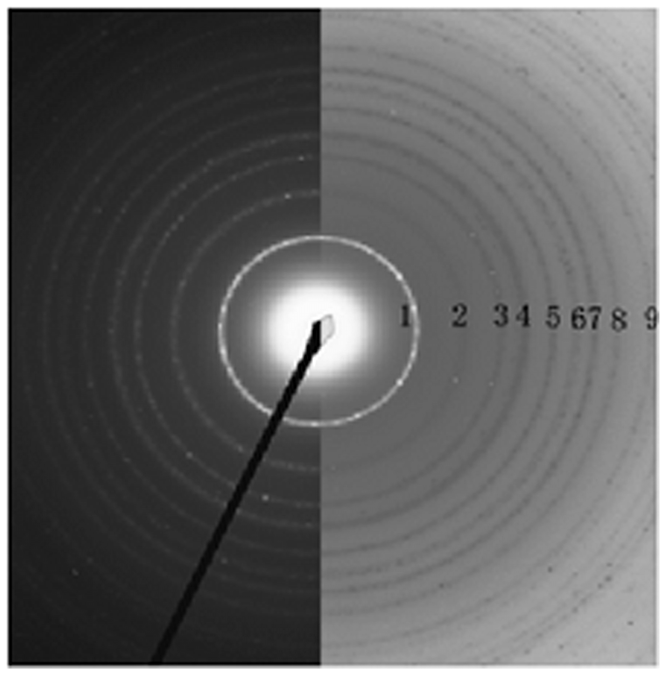
The diffraction rings of antase nanocrystals in details.

Figure 6 gave the electron diffraction (ED) pattern of some samples, the latter shows that the brightness and intensity of the polymorphic ring is weak, so the powder crystallized partially and was somewhat amorphous. For the samples that calcinated at 600°C 2 hours, the ED patterns were brighter and the intensity of the polymorphic ring is strong. As it shown, the samples were crystallized completely. Figure 7 shows the diffraction rings of antase nanocrystals in details. The diffraction rings are indexed in table 2 which showed the corresponding selected area electron diffraction (SAED). It confirmed the presence of crystal structure TiO2 according to JCPDS, card No∶ 21–1272.

**Table 2:** The Diffraction Rings Indexed according to JCPDS, card No∶ 21–1272.

Number	*d*	*h*	*k*	*l*
1	3.520 0	1	0	1
2	2.431 0	1	0	3
	2.378 0	0	0	4
	2.332 0	1	1	2
3	1.892 0	2	0	0
4	1.699 9	1	0	5
5	1.666 5	2	1	1
6	1.493 0	2	1	3
	1.480 8	2	0	4
7	1.364 1	1	1	6
8	1.337 8	2	2	0
9	1.279 5	1	0	7
	1.264 9	2	1	5

Table 3 shows the typical chemical composition of sample 1 after calcination at 400°C 2 hours, obtained from the EDS. As we can see from table 3, in sample 1, except Ti and O, other elements such as S, Cl and C are not detectable. So powders are pure TiO_2_. Other samples have the similar results.

**Table 3:** EDS of Sample 1 after Calcination at 400°C for 2 Hours.

Elt.	Line	Intensity (c/s)	Error 2-sig	Atomic %	Conc	Units	
O	Ka	60.83	2.014	64.081	37.356	wt.%	
Ti	Ka	556.14	6.089	35.919	62.644	wt.%	
				100.000	100.000	wt.%	Total

### Conclusions

In conclusion, we report that titania nanocrystals in anatase phase have been synthesized from hydrolysis of TiCl_4_ with sulfuric acid solution. The presence of a certain amount of H_2_SO_4_ promotes occurrence of anatase phase and inhibits the anatase-rutile transformation even at 600°C. After the powders calcinated at 600°C for 2 hours, some samples became completely anatase. Both the calcinations temperature and hydrolysis temperature have important effects on the primary particle size. The compositions of samples are pure anatase, without other elements. A new methodology is reported for preparing uniformly sized nanocrystals of the pure anatase phase that have a well-controlled particle size at controlled temperatures and compositions. The new process should have great potential in preparation of large amount of pure antase nanocrystals.
